# A new non-naked species of *Ptychostomella* (Gastrotricha) from Brazil

**DOI:** 10.3897/zookeys.289.4683

**Published:** 2013-04-12

**Authors:** M. Antonio Todaro

**Affiliations:** 1 Department of Life Sciences, University of Modena and Reggio Emilia, via Campi 213/d, I-41125, Italy

**Keywords:** Gastrotrichs, Brazil, São Paulo, meiofauna, biodiversity, taxonomy, new species, key

## Abstract

A new species of marine Gastrotricha from Brazil is described and discussed. *Ptychostomella lamelliphora* **sp. n.** is one of the several new taxa that were found during an extensive survey of the gastrotrich fauna carried out in 2002 and 2003 along the coastline of the State of São Paulo. The new species is unique in that it possesses cuticular ornamentations in the form of plate-like structures (scales) along the lateral borders of the body and two massive clusters of densely packed adhesive tubes on the ventral surface, near the ano-genital opening. Both these features appear to be adaptations to challenge the high energy waters that characterize the species’ microhabitat: the coarse sublittoral sand in the channel between the mainland and the largest island in the State, Ihlabela. Additionally, a key to the described *Ptychostomella* species of the world is provided.

## Introduction

The study is part of a larger research program aimed at shedding light on the diversity of marine invertebrates of the northern coasts of the State of São Paulo, Brazil (see Migotto and Tiago 1999). In the seminal works by Todaro and Rocha (2004a, b, 2005), faunistic and preliminary taxonomic data on the gastrotrich communities along the coastline comprised between Picinguaba to the north (at the Rio de Janeiro-São Paulo States border) and Praia Preta e das Choncas to the south were reported. Among the some 40 taxa found, the occurrence of several species new to science was highlighted. A recent article has dealt with one of such taxa (Todaro 2012); another interesting macrodasyidan in the family Thaumastodermatidae is here described.

## Methods

Survey along the northern coasts of the State of São Paulo took place between 20 April and 3 May 2002 and in September 2003. A general account on the visited locations are reported in Todaro and Rocha (2004a, 2005). Samples containing the new species were collected in 2002 from the sublittoral of Praia Grande and Beluga both on the Ilhabela side of the São Sebastião channel. Ilhabela is the largest island in the State and it is located just in front of the city of São Sebastião. Sandy substratum was collected by scuba diving using eight 500ml plastic jars, four per location. After collection, samples from each site were taken as soon as possible to the São Paulo University’s CEBIMar laboratory in São Sebastião. In the laboratory, the specimens were extracted daily with the narcotization-decantation technique using a 7% magnesium chloride solution within one week of collection. The supernatant was poured, without filtering, into 3.0 cm diameter plastic Petri dishes and scanned for gastrotrichs at 50 × under a Wild M8 stereomicroscope (see Todaro and Hummon 2008). Found gastrotrichs were mounted on glass slides and observed *in vivo* with Nomarski differential interference contrast optics using a Zeiss Axioscop 2 Plus microscope. During observation, the specimens were measured using an ocular micrometer and photographed with a Nikon Coolpix 995 digital camera (3.34 Mpixel). Some specimens were fixed overnight in a 1.0 M phosphate-buffered (pH 7.3) solution of paraformaldehyde, gluteraldehyde and picric acid, following Ermak and Eakin (1976), and stored for later SEM analysis. Gastrotrichs were rinsed in 0.2 M cacodylate buffer, dehydrated through a graded ethanol series, critical point-dried using CO_2_, mounted on aluminium stubs, sputter coated with gold-palladium and observed with a Philips XL 30 scanning electron microscope at the author’s Institution.

The description of the new species follows the convention of Hummon et al. (1993), whereas the position of some morphological characteristics along the body are given in percentage units (U) of total body length measured from anterior to posterior.

Granulometric analysis of the substrata was carried out according to Todaro et al. (2006). Mean grain size, sorting coefficient, kurtosis, and skewness were calculated by a computerized programme (Todaro 1992).

Abbreviations are as follows: PhIJ, pharyngeo-intestinal junction; TbA, adhesive tubes of the anterior series; TbL, adhesive tubes of the lateral series; TbV, adhesive tubes of the ventral series; TbP, adhesive tubes of the posterior series.

The rationale for the key to the ecological characteristics of the species, according to Hummon et al. (1992),is as follows: Frequency of a species from among a sample series (i.e., frequency of a species in samples collected in any given sampling trip) - Sparse, found in less than 10% of samples; occasional, found in 10–30% of samples; common, found in 30–60% of samples; usual, found in more than 60% of samples.

Abundance of a species among other species of a sample - Rare, less than 1% of a sample; scarce, 3–5% of a samples; numerous, 10–20% of a sample (often a sub-dominant); prevalent, more than 30% of a sample (usually dominant or co-dominant).

## Taxonomic account

### Order Macrodasyida Remane, 1925 [Rao & Clausen, 1970]. Family Thaumastodermatidae Remane, 1927. Subfamily Thaumastodermatinae Remane, 1927. Genus *Ptychostomella* Remane, 1926

#### Ptychostomella lamelliphora sp. n.

urn:lsid:zoobank.org:act:05285351-37C4-4343-9864-74B4C240C293

http://species-id.net/wiki/Ptychostomella_lamelliphora

Figs 1–4

##### Type locality.

Praia Grande on Ilhabela, State of São Paulo, Brazil (Lat. 23°51'S; Long. 45°25'W), at 3 m water depth in coarse (mean grain size, 0.51 mm), moderately well sorted (sorting, 0.85 mm) siliceous sand. Values of salinity, temperature and pH of the interstitial water at date of sampling 35.0‰, 23.2 °C and 7.90 respectively (Table 1).

**Figure 1.**
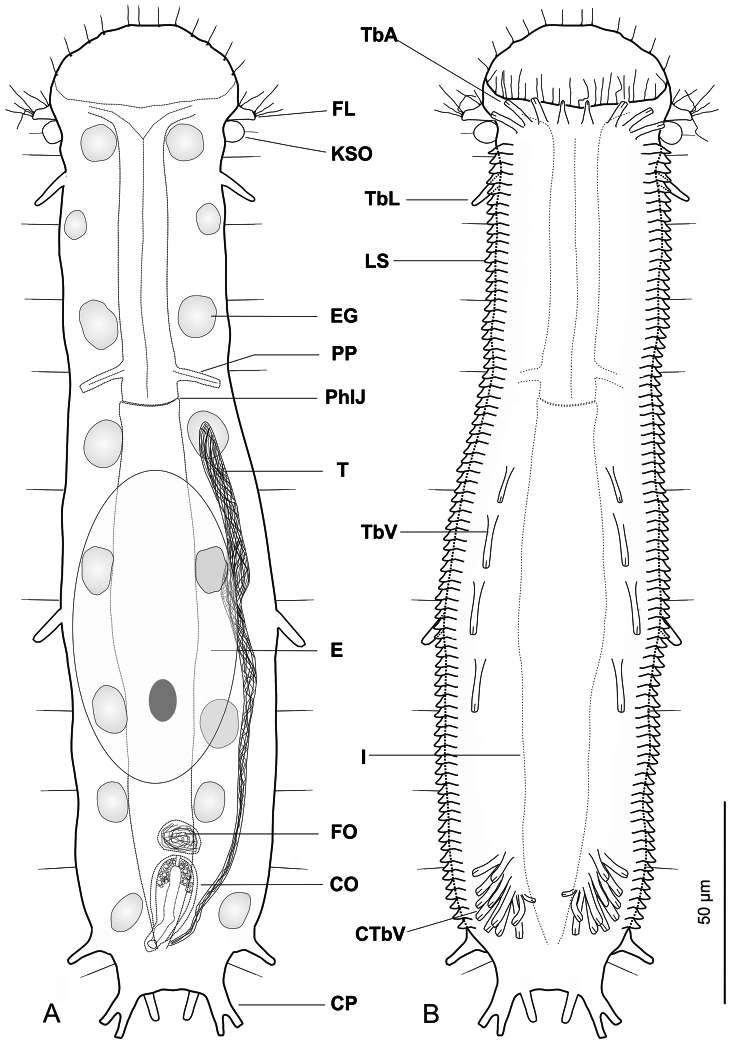
*Ptychostomella lamelliphora* sp. n. schematic drawings. **A** Habitus as seen from the dorsal side showing the internal anatomy **B** Habitus as seen from the ventral side. **CO** caudal organ **CP** caudal pedicle **CTbV** cluster of ventral adhesive tubes **E** egg **EG** epidermal gland **FL** fleshy lobe **FO** frontal organ **I** intestine **KSO** Knob-like sensory organ **LS** lamellate scales **PhIJ** pharyngeo-intestinal junction **Pp** pharyngeal pores **T** testicle **TbA** anterior adhesive tubes **TbL** lateral adhesive tubes **TbV** ventral adhesive tubes.

**Figure 2.**
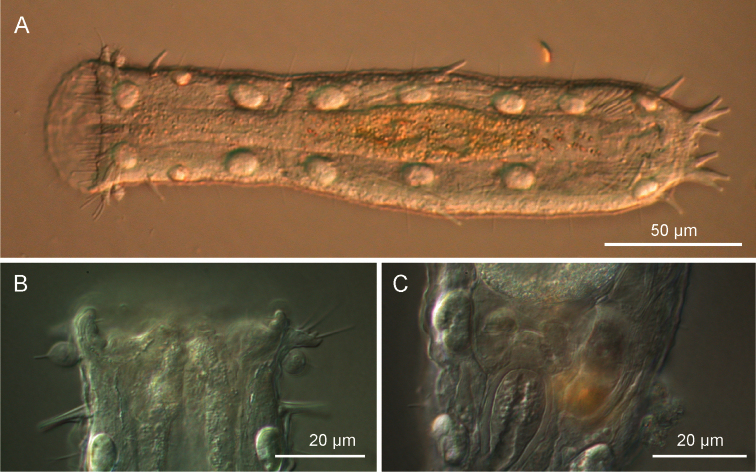
*Ptychostomella lamelliphora* sp. n. DIC photomicrographs. **A** habitus **B** close-up of the anterior region showing the knob-like sensory organs and the trapezoidal, fleshy lobes **C** Close-up of the posterior region of the trunk showing the caudal organ.

**Figure 3.**
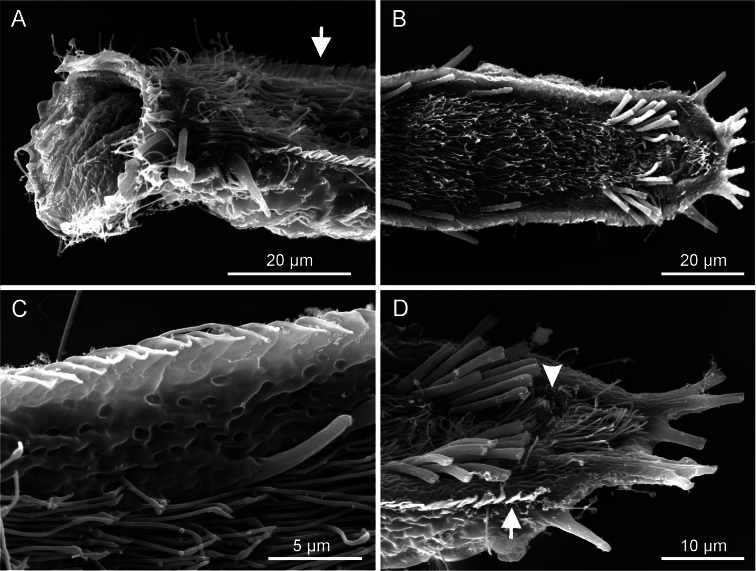
*Ptychostomella lamelliphora* sp. n. SEM photomicrographs ventral view. **A** close-up of the anterior region showing, among others, the plate-like-scales(arrow) **B** trunk region showing the locomotory ciliation and most of the tubular adhesive apparatus **C** close-up of the ventrolateral region of the trunk, showing the cuticle punctuated by shallow pits **D** close-up of the posterior region showing the two clusters of ventral adhesive tubes, the ano-genital opening (arrowhead) and the column of plate-like-scales.

**Figure 4.**
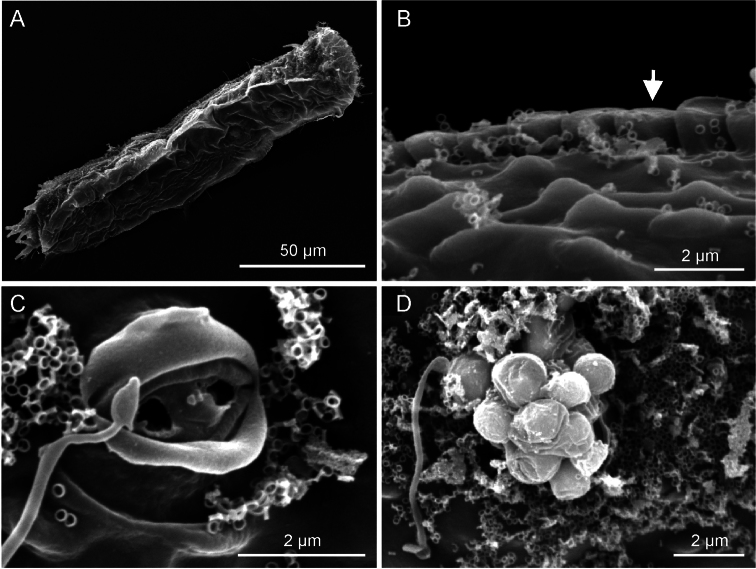
*Ptychostomella lamelliphora* sp. n.SEM photomicrographs, dorsal view. **A** habitus **B** close-up of the midtrunk region showing the plate-like scales (arrow) and the cuticle embossed with keel-like ornamentation **C** close-up of an epidermal gland pore **D** close-up of an epidermal gland secretion droplets.

**Table 1.** Sampling locations along the São Sebastião channel in the State of São Paulo, Brazil; geographic coordinates, date of collection, water depth and physical, chemical characteristics of the water and granulometric characteristics of the sediment.<br/>

**Variable**	**Locality**	
	**Praia Grande of Ihlabela**	**Beluga**
Geographic coordinates	23°51'S, 45°25'W	23°52'S, 45°26'W
Date of sampling	30/04/2002	30/04/2002
Salinity	35.0 ‰	35.2 ‰
Temperature	23.2 (°C)	22.4 (°C)
pH	7.90	7.93
Water Depth	3.0 m	6.0 m
Mean grain size and size class	0.95 phi<br/> coarse sand	0.55 phi<br/> coarse sand
Sorting and<br/> Sorting class	0.60 phi,<br/> moderately well sorted	0.46 phi<br/> well sorted
Skewness	0.23	1.25
Kurtosis	2.12	5.40

##### Type specimens.

Holotype, the adult specimen 250 µm long shown in Figure 2 (International Code of Zoological Nomenclature, Articles 73.1.1 and 73.1.4). After observation it was fixed in 95% ethanol and subsequently utilised for DNA extraction and 18S, 28S and CO I gene sequencing (GenBank accession number JF357643, JF357691 and JF432033 respectively, see Todaro et al. 2011, *Ptychostomella* sp1).

##### Material examined.

Eightadult specimens(including the holotype) collected by the author, five from the type locality and three from a Beluga a nearby location (see Table 1). Four specimens were observed alive and are not longer extant, while four were prepared for SEM survey and are kept in the meiofauna collection of the author (Ref. n. 2002-BR-01-02-05-06).

##### Ecology.

Frequency of occurrence: sparse, found only in sub-littoral sediment of two locations along the southern portion of the São Sebastião channel. Abundance: numerous in coarse sediment with little detritus of Praia Grande, scarce in coarse sediment rich in detritus of Beluga.

##### Diagnosis.

A *Ptychostomella* with an adult length to 250 µm; pharynx length to 74 µm, with pharyngeal pores at base. PhIJ at U37; body with almost parallel sides and short, bilobed caudum. Head bearing paired knob-like sensory organs and small, trapezoidal, fleshy lobes; eye spots missing; sensory hairs a few, forming lateral columns along the body, a fringe around the oral opening and loose tufts at the tip of the lobes; epidermal glands noticeable, eight per side, scattered along the length of the body. Cuticular covering generally smooth except for peculiar, subrectagular scales arranged in a column of on each ventrolateral side. Adhesive tubes: TbA, 4 per side, one slightly smaller, cone-shaped, in the middle at U9 and three lateral, rod-like, of equal size at U9-U10; TbL, 3 on each side, roughly of the same size, a small isolated one implanted anteriorly at U15, one in mid trunk region at U58 and one more robust near the base of the caudal lobes, at U90. TbV, up to 16 per side, four of the same size more or less evenly spaced, implanted along trunk region from U44 to U63, the remainder 10-12 forming a noticeable cluster at U83-U85. TbP, six in all, two medial and two on each of two paired caudal pedicles. Ventral locomotor cilia: a continuous field of transverse rows covering the entire surface except the ano-genital area. Reproductive system: testis on the right body side, caudal organ pyriform, frontal organ round filled with motile spermatozoa, a ripe egg dorsally in the mid-intestinal region.

##### Etymology.

The specific epithet *lamelliphora* (*lamella*, L, thin plateand *phero*, Gr., to bear)refers to the presence of the thin, plate-like scales along the ventrolateral body sides.

##### Description.

Description is mainly based on the holotypic specimen, 250 µm in total length. Pharynx 74 µm in length, measured from the posterior margin of the oral opening to the pharyngeo-intestinal junction, with pharyngeal pores near the base, at U34; pharyngeo-intestinal junction at U37. Head bearing paired knob-like sensory organs and small, trapezoidal, fleshy lobes; body robust, with side lines slightly widening to mid-trunk, then gradually narrowing to a short, bilobed caudum; widths of head\neck\trunk\caudal base 46\34\56\26 µm at U07\U25\U46\U93, respectively.

Oral hood slightly protruding anteriorly (to U08), with gently undulating borders. Sensory hair sparse, up to 7 µm in length, forming a fringe around the oral opening and loose tufts at the tip of the head’s lobes; a single hair emerges from each knob-like tentacle; other sensory hairs 9–14 µm in length form lateral and dorsolateral columns that are evenly spaced within columns but differ between columns. Eight pairs of noticeable epidermal glands are regularly spaced along the pharyngeal and intestinal region from U11 to U 87 with glands of the second and eighth pairs positioned somewhat more lateral; glands are round in shape and roughly of the same size (7–9 µm in diameter), except for the one of the second pairs, markedly smaller (4–6 µm). Each gland opens to the exterior via a well structured pore, the produced material is excreted in the form of small round droplets (see Figure 4C, D).

*Cuticular armature*. Body covering apparently smooth as typical of the genus, however on the ventrolateral sides, the cuticle generates subrectangular plates (scales), partially overlapping each other and tightly arranged in two columns running from U11 to U88. Scales, roughly of the same size (1.5–2.0 µm), protrude from the body and form bilateral structures that recall the lateral aerodynamic ‘mini-skirts’ of racing cars.

*Adhesive tubes*. TbA, 4 per side, inserting directly on the body surface at U9-U 10, one 4 µm in length medially and three 6–7 µm in length laterally. TbL, 3 per side (8–10 µm in length) inserting respectively at U15, U58 and U90. TbD, absent. TbV, up to 16 per side; 4 (8–11 µm in length) inserted singly along the intestinal region from U44 to U63 while the remaining 12 (4–11 µm in length) form an impressive cluster centered at U85 (cTbV; Figs 2A, 3B, D); tubes in the cluster originate singly and their number may slightly change from side to side. TbP, 3 per side, 2 (5–6 μm in length) at the end of each pedicle of the furcated caudum and the other one (6.5 μm in length) flanking each caudal pedicle medially.

*Ventral ciliation*. A continuous, dense field of cilia arranged in transverse rows that extend from the ventral border of the oral opening to the base of the caudal pedicles, being broadest at mid body and somewhat sparse in the ano-genital area at U90.

*Reproductive system*. testis on the right body side, caudal organ pyriform (10 × 21 μm), at U78; frontal organ bladder-like (10 μm in diameter) at U74.5; maturing eggs dorsal to the mid intestine.

##### Variability and remarks.

Length of the 4 living specimens ranged from 204 to 250 μm (mean = 230 μm, SD = 18 μm) all of them were mature (i.e., showed at least the testicles filled with sperm). The SEM prepared adult specimens resulted of smaller size (range 144–183 μm) even though size of these specimens appeared not dissimilar from the others under the dissecting microscope; these measurements fall well below the 5.5% length reduction allowed for fixed specimens (cf. Clausen 2004), but are in agreement with the shrinkage suffered by specimens of *Pseudostomella dolichopoda* Todaro, 2012 processed for SEM examination (cf. Todaro 2012). As SEM is being routinely utilized in species description, to avoid potential misidentification based on size, it would be interesting to explore the phenomenon of size reduction over a larger taxonomic spectrum. SEM prepared specimens showed some traits undetected or not present in living specimens. For instance, in SEM prepared specimens, *i*) the border of the oral hood appeared more scalloped than in living animals (cf. Figure 3A vs. Figure 2A), *ii*) the cuticle on the ventral area comprised between the ciliary field and the column of scales appeared punctuated by shallow pits (Figure 3C) and *iii*) the cuticle on the dorsal side appeared embossed with keel-like structures (Figure 4B). The adhesive tubes of the ventral series forming the clusters showed some variability in number, depending on individuals and on side of the body (e.g., see Figure 3B); the highest number of tubes, 12, was found in the cluster on the left side of two specimens including the holotype, while the lowest, 8 tubes, was found in the cluster on the right side of a specimen measuring 224 μm in total body length.

##### Taxonomic affinities.

The genus *Ptychostomella* was originally created to include small thaumastodermatid gastrotrichs whose body is enveloped by a smooth cuticle i.e., a cuticle that does not give rise to the typical scales and/or spines (e.g., ancres) found in other members of the family (Remane 1926). In a phylogenetic framework, the absence of such structures has been thought as a secondary reduction (cf. Todaro et al. 2011). While all of the known *Ptychostomella* species lack an armature typical of the family, at least 4 out of the 12 species described so far (Hummon and Todaro 2010) possess other kinds of cuticular ornamentations. *Ptychostomella lepidota* Clausen, 2000 bears scale-like cuticular elevation and *Ptychostomella orientalis* Lee & Chang, 2003 has the cuticular covering embossed with smooth hemispherical elevation (Clausen 2000; Lee and Chang 2003). A third species *Ptychostomella brachycephala* (Levi, 1954), originally affiliated to the genus *Platydasys*, possesses on each lateral side a column of rod-like papillae, and papillae are reported also for *Ptychostomella papillata* Lee and Chang 2003 (Lee and Chang 2003; Clausen 2004). Shape and arrangement of the cuticular ornamentations (i.e., subrectangular plates tightly arranged in two lateral columns) differentiate *Ptychostomella lamelliphora* n. sp. from all the four species reported above. The coexistence of knob-like sensory organs and of fleshy lobes on the head and the presence of most of TbV arranged in a bilateral clusters near the ano-genital opening may further distinguish the new species from all the previously known *Ptychostomella* species.

##### Conclusive remarks.

Adhesive tubes of the ventral series forming ‘feet’ or ‘clusters’ are not uncommon among members of the family Thaumastodermatidae (e.g., *Tetranchyroderma* and *Pseudostomella*) and they are present also in members of the genus *Ptychostomella* e.g., *Ptychostomella bergensis* Clausen, 1996 (Clausen 1996). However, what makes special the clusters of TbV present in the new species from Brazil is their bulkiness. To my knowledge no other gastrotrich species is known to posses bilateral clusters made up of such a high number of tubes. What is the adaptive advantage of such a formidable apparatus, and in species with fewer adhesive tubes, do they have the same function?

Adhesive and aptic structures are universally present among interstitial animals (Swedmark 1964) and it is possible to detect a relationship between the extent of the aptic apparatus and the energy of the environment water the species live in. For example, the gastrotrich species of the genus *Oregodasys* Hummon, 2008 that inhabit the coarse sediment typical of high energy waters are characterized by a formidable adhesive apparatus made up of tens of tubules (e.g., Rothe and Schmidt-Rhaesa 2010); high number of adhesive tubes also characterize species of the genus *Diplodasys* Remane, 1927 (e.g., Hummon and Todaro 2009), which often co-occur with *Oregodasys*. In this framework, it is natural to hypothesize that the massive clusters of adhesive tubules of *Ptychostomella lamelliphora* n. sp.are an adaptation that prevent the displacement of the animals by the strong currents that characterize the habitat. The high energy of these waters are indirectly indicated by the coarse sediment of the locus typicus in the São Sebastião channel (Table 1). In my view this working hypothesis is further supported by the presence in *Ptychostomella lamelliphora* n. sp. of the columns of scales along the ventrolateral sides whose function could be the reduction of the hydrodynamic turbulence around the body allowing a better bond of the gastrotrich to the sedimentary granules.

An alternative hypothesis could be that these structures play a role during reproduction e.g., used for sperm transfer or holding of the partner during cross fertilization. Only future TEM studies revealing ultrastructural difference between the ventral tubules clustering near the ano-genital opening and the genuine adhesive tubes (e.g., single tubes) could make the second hypothesis on this subject most plausible. A study of reproductive behaviour would also be revealing.

##### Taxonomic key.

Lee and Chang (2003)provided a useful taxonomic key to the species of the genus *Ptychostomella*; however, because one species has been transferred to this genus and the two additional ones have been described in the meanwhile (cf. Clausen 2004; Lee et al. 2009) a revised key seems necessary. The following key is based upon characters visible under light microscopy.

**Table:** 

1	dorsal surface smooth	2
–	other	11
2	lateral margins smooth	3
–	other	10
3	eyespots present	*Ptychostomella ommatophora* Remane, 1927
–	other	4
4	head with knob-like or club-shaped sensory organs	5
–	other	8
5	head with paired club-shaped sensory organs	*Ptychostomella helane* Roszczak, 1939
–	head with paired knob-like sensory organs	6
6	adhesive tubes between the caudal pedicles present	7
–	adhesive tubes between the caudal pedicles absent	*Ptychostomella tyrrhenica* Hummon, Todaro & Tongiorgi, 1993
7	4 (2 + 2) adhesive tubes between the caudal pedicles	*Ptychostomella mediterranea* Remane, 1927
–	up to 10 (5 + 5) adhesive tubes between the caudal pedicles	*Ptychostomella higginsi* Clausen, 2004
8	with some of the TbV forming a pair of clusters or ventral feet (4 + 4 tubes each)	*Ptychostomella bergensis* Clausen, 1996
–	without cluster of TbV	9
9	TbV evenly space along the intestinal region	*Ptychostomella jejuensis* Lee, Hwang & Chang, 2009
–	TbV gathered in the first third of the intestinal region	*Ptychostomella pectinata* Remane, 1926
10	each lateral side bearing a column of rod-like papillae	*Ptychostomella brachycephala* (Levi, 1954)
–	each lateral side bearing a column of subrectangular scales	*Ptychostomella lamelliphora* n. sp.
11	cuticular covering bearing scale-like elevantions	*Ptychostomella lepidota* Clausen, 2000
–	other	12
12	cuticular covering embossed with smooth hemispherical elevantions	*Ptychostomella orientalis* Lee & Chang, 2003
–	dorsal surface with terrace-shaped cuticular protrusions on head and numerous papillae with sensory hair(s)	*Ptychostomella papillata* Lee & Chang 2003


## Supplementary Material

XML Treatment for Ptychostomella lamelliphora
